# *Spondias purpurea* L. (Anacardiaceae): Antioxidant and Antiulcer Activities of the Leaf Hexane Extract

**DOI:** 10.1155/2017/6593073

**Published:** 2017-10-26

**Authors:** Cynthia Layse Ferreira de Almeida, Samara Alves Brito, Temístocles Italo de Santana, Henrique Bandeira Alves Costa, Carlson Helder Reis de Carvalho Júnior, Márcia Vanusa da Silva, Lécio Leone de Almeida, Larissa Araújo Rolim, Vanda Lucia dos Santos, Almir Gonçalves Wanderley, Teresinha Gonçalves da Silva

**Affiliations:** ^1^Postgraduate Program of Pharmaceutical Sciences, Universidade Federal de Pernambuco, Recife, PE, Brazil; ^2^Department of Antibiotics, Universidade Federal de Pernambuco, Recife, PE, Brazil; ^3^Department of Biochemistry, Universidade Federal de Pernambuco, Recife, PE, Brazil; ^4^Department of Biological Sciences, Universidade Regional do Cariri, Crato, CE, Brazil; ^5^Analytical Center of Drugs, Medicines and Food, Universidade Federal do Vale do São Francisco, Petrolina, PE, Brazil; ^6^Department of Pharmaceutical Sciences, Universidade Estadual da Paraíba, Campina Grande, PB, Brazil; ^7^Department of Physiology and Pharmacology, Universidade Federal de Pernambuco, Recife, PE, Brazil

## Abstract

*Spondias purpurea* is used in folk medicine to treat diarrhea and diuresis. The objective of this study was to evaluate the phytochemical profile and antioxidant and antiulcer activities of the hexane extract of the leaves of *S. purpurea* (SpHE). Phytochemical profile was evaluated via thin layer chromatography (TLC) and HPLC. SpHE was screened for antioxidant activities using DPPH, ABTS, FRAP, and phosphomolybdenum assays. To determine its antiulcer properties, animals were pretreated with injured control, lansoprazole, ranitidine, carbenoxolone, or SpHE (12.5, 25, and 50 mg/kg) and were screened; acute ulcers were induced by HCl/ethanol, absolute ethanol, and nonsteroidal anti-inflammatory drug (NSAID). TLC revealed the presence of flavonoids, whereas HPLC analysis showed the presence of caffeic acid and epigallocatechin. The phenolic compounds and *in vitro* assays showed antioxidant activity. After gastric ulcer induction by using HCl/ethanol, SpHE reduced the area of ulcerative lesions by 82, 91, and 88%, respectively. In ethanol, SpHE reduced the area of ulcerative lesions by 77, 93, and 92%, respectively. In the NSAID, the percentages of protection were 70, 76, and 78%, respectively. SpHE promoted the minimization of ulcers, increased the levels of reduced glutathione, and decreased tumor necrosis factor. *S. purpurea* has antioxidant and antiulcer properties.

## 1. Introduction

Peptic ulcers involve inflammatory or necrotizing conditions that can reach the mucous membranes of the esophagus, stomach, and duodenum and are caused by an imbalance between defensive and aggressive factors in the mucosa [1, 2]. These lesions affect approximately four million people worldwide, and 10–20% of cases develop complications, with a mortality rate of 10–40%. Symptoms include epigastric pain, bleeding, obstruction, and perforation, which may progress to death [3–6].

The development of ulcerative lesions occurs due to an imbalance between the aggressive and the defensive factors of the gastric mucosa [7]. The aggressive factors result from the union of endogenous factors, such as HCl, pepsin, biliary reflux, lipid peroxidation, and the formation of reactive oxygen species (ROS), and exogenous factors related to contemporary lifestyles, such as the excessive use of ethanol, indiscriminate use of nonsteroidal anti-inflammatory drugs (NSAIDs), stress, smoking, and infection by *Helicobacter pylori* [8–10]. Cytoprotective factors include the mucobicarbonate barrier, mucin secretion, surface phospholipids, prostaglandins (PGs), nitric oxide (NO), mucosal blood flow, cell renewal, growth factors, and antioxidant enzymes [8, 9, 11, 12].

Oxidative stress is related to the impairment of cellular viability and results in the activation of repair mechanisms, neutrophil accumulation, the production of proinflammatory cytokines, the generation of ROS, the reduction of blood flow in the mucosa, and apoptosis and/or necrosis, all of which are determining factors for the appearance of gastric lesions [13].

Despite the availability of effective therapies, such as H_2_ receptor antagonists and proton pump inhibitors, there is still no absolute cure for this disease. In addition, side effects and drug interactions have been related to the long-term use of such agents [14]. Long-term use of proton pump inhibitors may be associated with ineffectiveness of different drug regimens, and even resistance to these drugs has been emerging [15]. Therefore, it is essential to research and develop new therapeutic alternatives that demonstrate good effectiveness with fewer side effects, as well as therapies for the improvement of ulcer healing and the prevention of disease recurrence.

Brazil is a country with the greatest plant diversity in the world and has about 20% of the total number of species on the planet, but medicinal plants form Brazil are used with little or no proof of their pharmacological properties [16].

The Anacardeaceae family comprises approximately 70 genera and 600 species, consisting mainly of trees and shrubs typical of tropical, subtropical, and temperate regions of the world [17]. Of these, 14 genera and 57 species occur in Brazil, of which 14 species are endemic to the caatinga, an exclusive biome of Brazil with great biological patrimony. The genus *Spondias* (Anacardiaceae) consists of approximately 14 species distributed worldwide, cultivated, and marketed due to their fruits that are consumed raw or processed into pulps, juices, and other food products [17–19].

*Spondias purpurea* L. (Anacardiaceae), popularly known as “serigueleira,” is a plant native to the subtropical semiarid forests of Mesoamerica, Peru, and Brazil. It produces smooth, bright green, yellow, orange, or red oval fruits up to 5.5 cm in length and with a mass ranging from 12 to 28 g, which are consumed in both raw and processed forms [17, 20].

In folk medicine, various parts of the *S. purpurea* are used to treat gastric disorders, as antidiarrheals and as diuretics [17, 21, 22]. These activities may be related to the antioxidant properties already reported for this species and for the genus [23, 24] and to the phenolic compound content present in *S. purpurea* [17].

Phenolic compounds are important chemical constituents of plants with antiulcerogenic activity. Their antioxidant properties and ability to reduce lipid peroxidation lead to the prevention and/or delay of cellular necrosis and improve the vascularization of the affected region [25].

Among the isolated metabolites of *S. purpurea*, studies show that chemical composition of the leaf oil to contain *β*-caryophyllene, *δ*-cadinene, torreyol, and T-muurolol [26]. In this species, carotenoid pigments lutein and zeaxanthin [27], phenolic acids (gallic acid, chlorogenic acid), and flavonol O-glycosides of quercetin, kaempferol, kaempferide, and rhamnetin were extracted from the fruits [17]. In the volatile composition of the fruit, the pulp of the fresh fruit made it possible to identify ketones, alcohols, aldehydes, esters, and terpenic hydrocarbons in the headspace, and the major compounds identified were hexanal, trans-2-hexenal, 3-hexen-1-ol, 2-hexen-1-ol, ethyl acetate, and hexyl acetate [28]. In *S. purpurea* fruits, the phytochemical screening revealed the presence of phenols, quercetin, chlorogenic acid, citric acid, tannins, anthraquinones, anthrone, coumarins, triterpenoids, and steroids (peel and seeds); quercetin, anthocyanins, proanthocyanidins, and flavonoids (peel); and saponins, leucoanthocyanidins, catechins, and flavanones (seeds) [29]. Besides, galactose, arabinose, mannose, xylose, rhamnose, and uronic acids constituted the polysaccharide gum of *S. purpurea* [30].

Although several studies had been described for the fruits of *S. purpurea*, we did not find in the literature any work about the chemical composition and antiulcer activity of the extracts of the leaves, though this is the part used in folk medicine for treating gastric disorders. In view of this, we decided to investigate the chemical composition and pharmacological activities of the hexanic extract obtained from *S. purpurea* leaves.

## 2. Materials and Methods

### 2.1. Plant Material and Preparation of the Extract

The plant material used was the leaves of the species *S. purpurea* (Anacardiaceae) collected from Alhandra, Paraíba, Brazil (7°19′54.8″S, 34°57′39.5″W), in June 2014. A representative sample of this species was deposited at the Dárdano de Andrade Lima Herbarium of the Agronomic Institute of Pernambuco (IPA) (registration number 89986). The leaves were air dried in an oven with forced air circulation at 40°C and powdered. Thereafter, the plant material (600 g) was subjected to cold exhaustive maceration with three separate solvents (6000 mL) of differing polarities (hexane, ethyl acetate, and ethanol) under agitation for three consecutive periods of 72 h intervals each. The extracts were filtered and the solvents were completely removed with the aid of a low-pressure rotary evaporator. Then, a pharmacological screening for the three extracts at different doses was performed (unpublished data). In the evaluation of the results, it was possible to observe that among the three extracts, the hexanic extract presented the best antiulcer activity at lower doses in relation to the other extracts. In addition, the hexane extract presented a satisfactory yield (10%, *w*/*v*), which was superior to the other extracts, justifying its choice to study.

### 2.2. Animals

Wistar rats (180–250 g) and Swiss mice (25–35 g) of both sexes were obtained from the Department of Physiology and Pharmacology and the Department of Antibiotics, respectively, of the Federal University of Pernambuco (UFPE), Pernambuco, Brazil. The animals were maintained under standard environmental conditions (12 h dark/light cycle) and temperature (22 ± 2°C). Water and industrialized dry food (Presence, Purina, Brazil) were provided ad libitum. The animals were maintained in cages with raised wide mesh floors to prevent coprophagy. All the experimental protocols were approved by the Animal Experimentation Ethics Committee of the UFPE (number 23076.013615), in accordance with the National Institute of Health's Guide to the Care and Use of Laboratory Animals.

### 2.3. Thin Layer Chromatography

The presence of secondary metabolite groups in the SpHE was assessed using TLC, and the following specific chemical developers were used: flavonoids, cinnamic derivatives, phenylpropanoglycosides (NEU reagent and ferric chloride reagent), alkaloids (Dragendorff reagent), saponins (vanillin/sulfuric acid reagent), condensed proanthocyanidins, and leucoanthocyanidin (vanillin/HCl reagent) [31–33].

### 2.4. HPLC Analysis

To determine the chromatographic profile of SpHE, a LC-20 liquid chromatograph UFLC system (Shimadzu, Tokyo, Japan) was utilized, controlled by LC Software Solution 1.0 (Shimadzu, Tokyo, Japan). The HPLC contained a LC-20ADVP quaternary pump system, DGU-20A degasser, SPD-20AVP PDA detector, CTO-20ASVP furnace, SIL-20ADVP autosampler, and SCL-20AVP controller coupled with a diode array detector (DAD). The mobile phase consisted of solvent A (0.01% trifluoroacetic acid solution in ultrapure water) and solvent B (100% acetonitrile). The gradient conditions were as follows: 0–40 min 90–60% A, 40–50 min 60% A, and 50–60 min 60–90% A. A C18 Hypersil column was used (250 × 4.6 mm; 5 *μ*m; Thermo Fisher Scientific, Runcorn, United Kingdom) at 30°C. Each assay was performed in triplicate. For each assay, 50 *μ*L of sample was injected; the flow rate was 0.8 mL/min; and chromatograms were recorded at 320 nm. Authentic markers for the chromatographic comparison of data were supplied by Sigma-Aldrich Chemie (Steinheim, Germany) for HPLC analysis with purity ≥ 98%. These included apigenin, borneol, caffeic acid, catechin, chlorogenic acid, chrysin, ellagic acid, epicatechin, epigallocatechin, fisetin, gallic acid, gallocatechin, kaempferol, lupeol, myricetin, naringenin, p-coumaric acid, protocatechuic acid, quercetin, quercetin 3*β*, resveratrol, rutin, scopoletin, and tannic acid.

### 2.5. In Vitro Antioxidant Activity: Determination of Total Phenolic Content

The total phenolic content (TPC) of the SpHE was determined using Folin–Ciocalteu (FC) reagent, as described by Li et al. [34]. Gallic acid (0–500 mg/L) was used to calibrate a standard curve (triplicate). The results are expressed as mg of GA equivalents/g extract (mg GAE/g).

### 2.6. In Vitro Antioxidant Activity: Phosphomolybdenum Assay

The antioxidant activity of the SpHE was determined via the phosphomolybdenum method based on spectrophotometric determination of the reduction of Mo^4+^ to Mo^5+^, with the subsequent formation of Mo^5+^ phosphate, which has a maximum absorption at 695 nm [35]. The total antioxidant activity (TAA) was expressed relative to ascorbic acid and calculated by using the following formula: % TAA = (*A*_s_ − *A*_c_) × 100/(*A*_a_ − *A*_c_), where *A*_s_ is the absorbance in the presence of the extract, *A*_c_ is the control absorbance (white: without extract), and *A*_a_ is the absorbance of the ascorbic acid.

### 2.7. In Vitro Antioxidant Activity: Ferric Ion Reducing Power (FRAP) Assay

This assay was performed according to the method described by Benzie and Strain [36], which is based on the reduction of ferric tripyridyltriazine complex to its dark blue ferrous form, in the absence and presence of antioxidants. The results are expressed as Trolox equivalent antioxidant capacity (TEAC) values, calculated with respect to the original FRAP in mmol Trolox/g.

### 2.8. In Vitro Antioxidant Activity: 2,2-Azino-bis-3-ethylbenzothiazoline-6-sulfonic Acid (ABTS) Assay

For this test, the methodology described by Re et al. [37] was used. Initially, the radical ABTS^•+^ was formed from the reaction of 7 mM ABTS stock solution with 140 mM potassium persulfate. All experiments were carried out in triplicate. The percentages of oxidative inhibition were calculated and plotted as a function of the reference antioxidant concentration (Trolox) and expressed as TEAC (*μ*M).

### 2.9. In Vitro Antioxidant Activity: 2,2-Diphenyl-1-picrylhydrazyl (DPPH) Radical Scavenging Activity

Free radical sequestering activity was measured via hydrogen donation using the stable radical DPPH [38]. The percentage of inhibition (I%) was calculated using the following equation: I% = [(Abs_0_ − Abs_1_)/Abs_0_] × 100, where Abs_0_ is the absorbance of the control and Abs_1_ is the absorbance in the presence of the test compound.

### 2.10. Antiulcerogenic Activity: HCl/Ethanol-Induced Ulcer

Mice were divided into six groups (*n* = 6). Each mouse was fasted for 18 h prior to receiving an oral dose of an aqueous solution containing 1% Tween 80 aqueous solution (injured control (CL) (10 mL/kg)), lansoprazole 30 mg/kg (a proton pump inhibitor), or SpHE (at doses of 12.5, 25, and 50 mg/kg body weight). After 50 min, all the animals received 0.3 M HCl/ethanol 60% solution (1 mL/150 g) orally to induce acute gastric lesions, according to the method proposed by Mizui and Douteuchi [39], with modifications. The animals were killed with CO_2_ gas 1 h after the induction of gastric lesions. The stomachs were removed and opened along the greater curvature line and fixed between two glass plates. The ulcerative lesion index (ULI) was calculated according to the methodology described by Szelenyi and Thiemer [40].

### 2.11. Antiulcerogenic Activity: Ethanol-Induced Ulcer

This experiment was carried out as described by Morimoto et al. [41], with modifications. After 16 h of fasting, rats (*n* = 6/group) were orally administered an aqueous solution containing 1% Tween 80 (injured control (CL)), lansoprazole (30 mg/kg), or SpHE (12.5, 25, and 50 mg/kg). After 60 min, all groups were administered 4 mL/kg of absolute ethanol orally for gastric ulcer induction. The animals were killed with CO_2_ gas 1 h after the induction of gastric lesions. The stomachs were removed and photographed, and the surface area of the gastric lesion (ULA) was determined using computerized planimetry (ImageJ Software). The data are expressed in mm^2^.

### 2.12. Antiulcerogenic Activity: NSAID-Induced Ulcer

Wistar rats (*n* = 6/group), after 18 h of fasting, were orally treated with CL (injured control, 10 mL/kg), ranitidine 60 mg/kg (an H_2_ receptor antagonist), or SpHE (12.5, 25, and 50 mg/kg). Sixty minutes after the treatment, indomethacin (30 mg/kg) was administered subcutaneously to induce gastric lesions, according to the methodology described by Djahanguri [42], with modifications. Six hours after the administration of indomethacin, the animals were killed, and the stomachs were removed for the determination of gastric lesions, as previously described.

### 2.13. Histopathological Examination of Gastric Mucosa

Samples of the gastric mucosa obtained after treatments were preserved in 10% formaldehyde buffer. Immediately, they were washed in buffer, dehydrated in an increasing alcohol concentration series, and fixed in historesin glycol methacrylate (Historesin Leica, Leica Biosystems GmbH, Nussloch, Germany). Ultrathin sections (4 *μ*m) were obtained using a microtome (model RM 2245, Leica Biosystems GmbH, Nussloch, Germany) and subjected to hematoxylin and eosin (HE) staining. Two slides were prepared for each animal, and all of them were examined under a light microscope (model DM500, Leica Biosystems GmbH, Nussloch, Germany), photographed using Leica camera (model EC3, Leica Biosystems GmbH, Nussloch, Germany) attached to the microscope, and then histopathologically analyzed using Leica Application Suite (LAS) EZ microscope software (Leica Biosystems GmbH, Nussloch, Germany) [43, 44].

### 2.14. Measurement of Gastric Mucosal Reduced Glutathione (GSH)

The levels of GSH in the gastric mucosa after treatment with HCl/ethanol, absolute ethanol, and NSAID were determined using the method developed by Sedlak and Lindsay [45]. The concentrations of nonprotein sulfhydryl groups were expressed in *μ*g of GSH/mg of protein.

### 2.15. Measurement of Tumor Necrosis Factor-*α* (TNF-*α*) in Gastric Mucosa

To quantify cytokine levels, stomach tissue homogenates obtained from the experimental models were centrifuged for 10 min at 350 ×g and the supernatant was stored at −40°C until analysis. The concentrations of TNF-*α* were determined by enzyme-linked immunosorbent assay (ELISA) according to the manufacturer's instructions (eBioscience, San Diego, CA, USA). Results are expressed as pg/mL.

### 2.16. Measurement of Gastric Mucosal Nitric Oxide (NO)

The nitrite concentration in the stomach homogenate obtained was used as an index of nitric oxide production and was measured using the Griess reaction. Briefly, 50 *μ*L of each sample and 50 *μ*L of Griess reagent were placed in a 96-well microtiter plate and incubated at room temperature (22°C) for 10 min, while being protected from light. The absorbance was measured at a wavelength of 560 nm using a microplate reader, and nitrite concentration was determined by comparing the sample absorbance to a standard curve for sodium nitrite. The experiments were performed in triplicate, and the results are expressed in *μ*M [46].

### 2.17. Statistical Analysis

Values are expressed as mean ± standard deviation (SD). Statistical significance between groups was determined using a one-way analysis of variance (ANOVA) followed by Tukey's tests, with *p* < 0.05 indicating significance. All statistical analyses were performed using GraphPad Prism 7.0 (GraphPad Software Inc., La Jolla, CA, USA).

## 3. Results

### 3.1. Thin Layer Chromatography

Through phytochemical prospecting, the presence of flavonoids was observed. However, the presence of saponins, alkaloids, condensed proanthocyanidins, and leucoanthocyanidin was not observed.

### 3.2. HPLC Analysis

**Figure 1:**
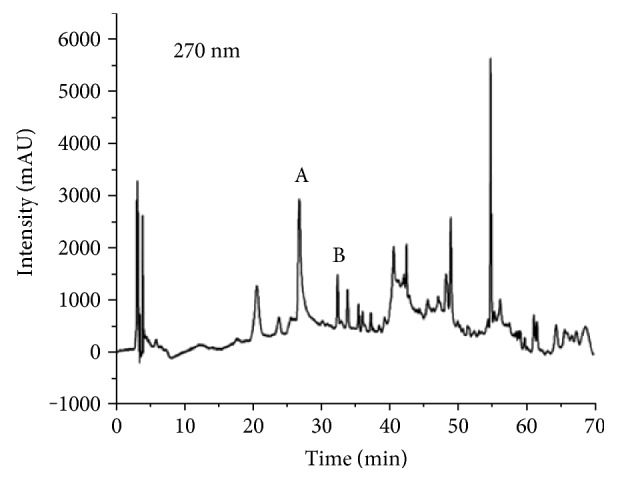
HPLC chromatograms of the hexane extract from leaves of *Spondias purpurea*. A, caffeic acid (26 min); B, epigallocatechin (32.25 min).

From the chromatographic analysis of the extract, it was possible to identify two compounds present by comparing the similarities of the retention times and UV absorption spectra with the corresponding values in the literature and to quantify them: caffeic acid (A) eluted after 26 min (8.60 *μ*g/mL ± 2.30) and epigallocatechin eluted after 32.5 min (3.01 *μ*g/mL ± 1.02) (Figure 1).

### 3.3. In Vitro Antioxidant Activity

In the quantification of the total phenolic compounds, the content found was 107.36 ± 1.82 mg GAE/g SpHE. In the phosphomolybdenum method, TAA was calculated in 13.10 ± 1.40%, in relation to ascorbic acid (activity considered 100%). In FRAP, the antioxidant capacity was measured based on the ability to reduce Fe^3+^ ions in Fe^2+^, and the result was 278.30 mmol Trolox/g SpHE. The antioxidant potential by the ABTS method was determined from the interpolation of the absorbance of the sample equivalent to the calibration curve constructed from the Trolox standard. TEAC was 114.44 ± 26.94, 116.67 ± 33.33, and 271.11 ± 31.68 mmol Trolox/g SpHE after 6, 30, and 60 minutes, respectively.  Table 1 shows the percentage of sequestration of DPPH radicals in different concentrations (31.25–1000 *μ*g/mL) of SpHE and EC_50_ = 546.67 *μ*g/mL.

### 3.4. Gastric Ulcer Induction by HCl/Ethanol

**Figure 2:**
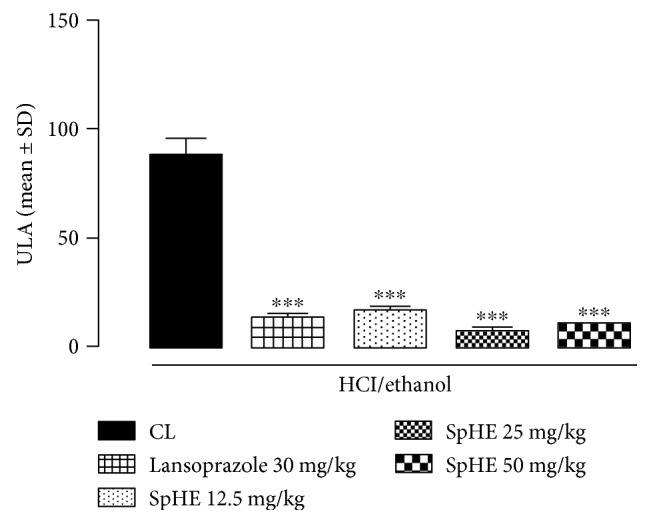
Effect of oral administration of SpHE on gastric lesions induced by HCl/ethanol in Swiss mice. Animals were treated orally with 1% Tween 80 aqueous solution (injured control (CL)), lansoprazole (30 mg/kg), and SpHE (12.5, 25, and 50 mg/kg). ULI: ulcerative lesion index. Results are expressed as mean ± SD (*n* = 5–6). ANOVA followed by Tukey's test, ^∗∗∗^*p* < 0.001 when compared with CL.

**Figure 3:**
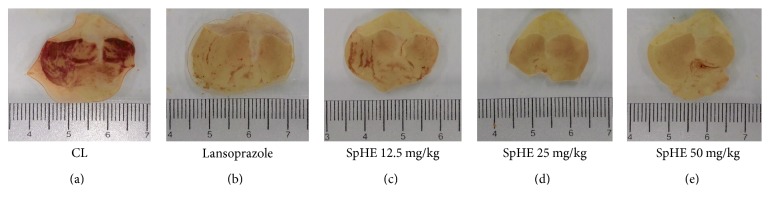
Effect of different doses of SpHE on the severity of gastric lesion (gross examination) examined in HCl-/ethanol-induced gastric ulceration model. These photographs are typical of such tissues.

In the HCl/ethanol-induced gastric ulcer model, SpHE led to a significant increase in protection (lesion areas: 16.12 ± 2.59, 7.50 ± 0.81, and 10.20 ± 0.18 mm^2^, resp.) when compared to that after injured control (CL) (87.17 ± 8.35 mm^2^), corresponding to 82, 91, and 88% of lesion area inhibition for doses of 12.5, 25, and 50 mg/kg, respectively. Lansoprazole (30 mg/kg) significantly reduced the gastric lesions by 85% (13.12 ± 1.27 mm^2^) (Figure 2). These results could be better visualized in a gross examination of the gastric mucosa. In animals pretreated with CL, severe lesions were observed with extensive visible hemorrhagic necrosis of the gastric mucosa. Only mild lesions of the gastric mucosa were observed in the other groups, as such abrasions were already present in animals pretreated with lansoprazole or SpHE (Figure 3).

### 3.5. Gastric Ulcer-Induction by Ethanol

**Figure 4:**
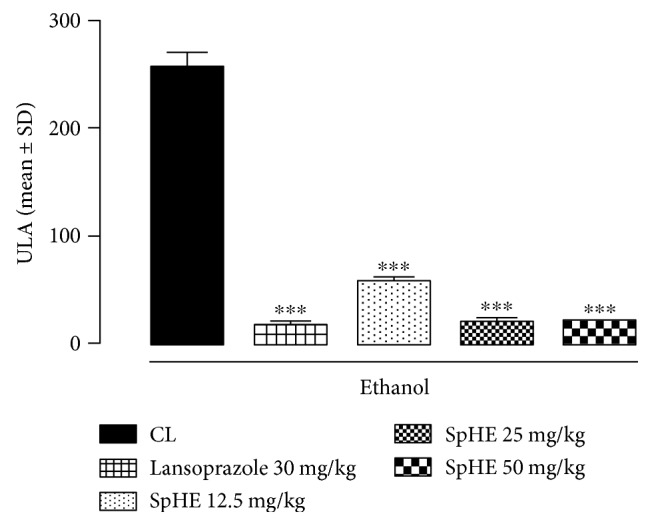
Effect of SpHE on gastric lesions induced by absolute ethanol in Wistar rats. Animals were treated orally with 1% Tween 80 aqueous solution (injured control (CL)), lansoprazole (30 mg/kg), and SpHE (12.5, 25, and 50 mg/kg). Ulcerative lesion area. Values represent the mean ± SD (*n* = 6). ANOVA followed by Tukey's test, ^∗∗∗^*p* < 0.001 when compared with CL.

**Figure 5:**
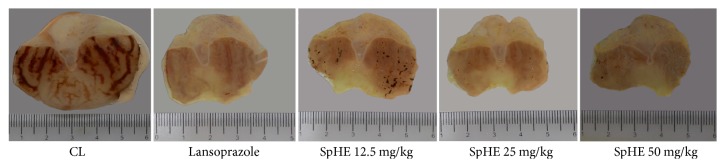
Effect of different doses of SpHE on the severity of gastric lesion (gross examination) examined in ethanol-induced gastric ulceration model. These photographs are typical of such tissues.

As shown in Figures 4 and 5, the administration of ethanol caused extensive damage to the gastric mucosa with hemorrhagic erosions seen in the injured control (CL) (254.60 ± 14.86 mm^2^). Oral administration of SpHE (12.5, 25, and 50 mg/kg) and lansoprazole significantly reduced the lesion area to 57.20 ± 4.81, 20.20 ± 2.75, 18.86 ± 1.78, and 17.22 ± 1.62 mm^2^, respectively, which corresponds to a percentage of inhibition of 78, 92, 93, and 93%, respectively.

### 3.6. Gastric Ulcer-Induction by NSAID

**Figure 6:**
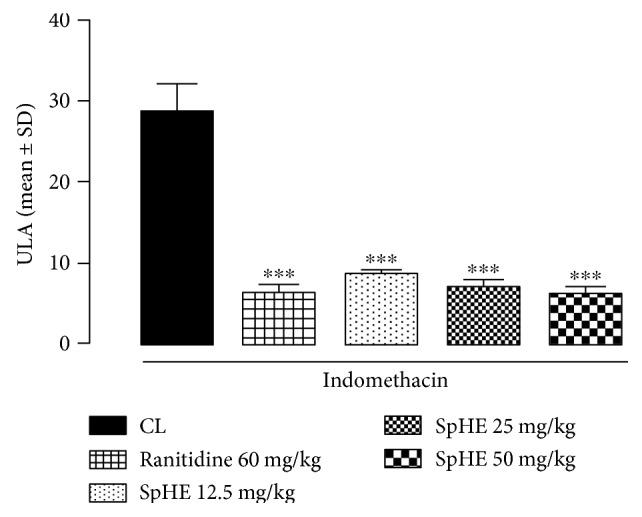
Effect of SpHE on gastric lesions induced by NSAID in Wistar rats. Animals were treated orally with 1% Tween 80 aqueous solution (injured control (CL)), ranitidine (60 mg/kg), and SpHE (12.5, 25, and 50 mg/kg). Ulcerative lesion area. Values represent the mean ± SD (*n* = 6). ANOVA followed by Tukey's test, ^∗∗∗^*p* < 0.001 when compared with CL.

**Figure 7:**
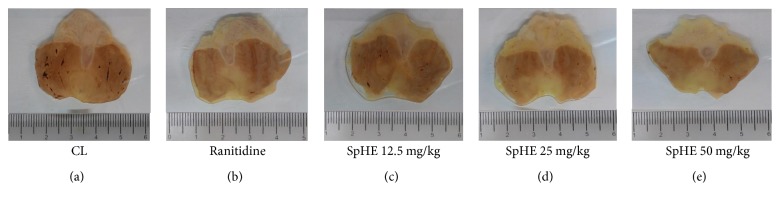
Effect of different doses of SpHE on the severity of gastric lesion (gross examination) examined in NSAID-induced gastric ulceration model. These photographs are typical of such tissues.

According to the results obtained in this model, it was observed that SpHE (12.5, 25, and 50 mg/kg) and ranitidine (60 mg/kg) significantly reduced the lesion area by 70, 75, 78, and 77% (8.70 ± 0.66, 7.04 ± 0.78, 6.19 ± 0.85, and 6.38 ± 0.98 mm^2^, resp.), when compared to the reduction after injured control (CL) (28.67 ± 3.38 mm^2^) (Figure 6). These results could be better visualized in a gross examination of the gastric mucosa (Figure 7).

### 3.7. Histopathological Examination of Gastric Mucosa

**Figure 8:**
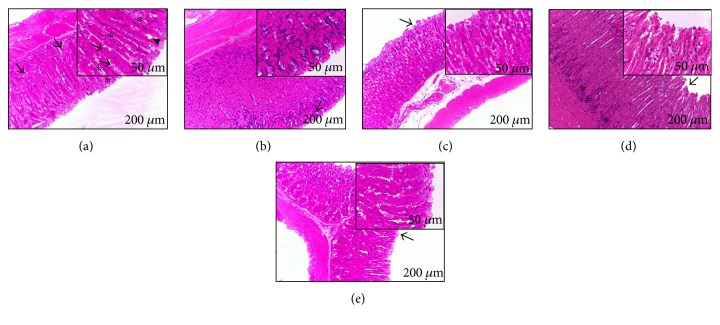
Histological analysis of the gastric mucosa of animals submitted to the induction of ulcer with HCl/ethanol. Animals were pretreated with 1% Tween 80 aqueous solution: injured control (a), lansoprazole (b), and SpHE 12.5 mg/kg (c), 25 mg/kg (d), and 50 mg/kg (e). Observed in (a): evidence of disorganization of the columnar simple epithelium in the gastric pits (enlarged detail, arrowhead) and gastric glands (arrows), congestion of the blood capillaries (enlarged detail, asterisk), and necrosis of the gastric mucosa cells (enlarged detail); in (b): preserved columnar simple epithelium in the gastric pits (enlarged detail) and well-preserved gastric glands (arrow); in (c): nonpreserved gastric mucosa with exfoliation of the simple columnar epithelium (enlarged detail); in (d): gastric mucosa with slight focal exfoliations of the simple columnar epithelium and preserved gastric mucosa (asterisk); and in (e): preserved gastric mucosa and simple columnar epithelium (enlarged detail).

**Figure 9:**
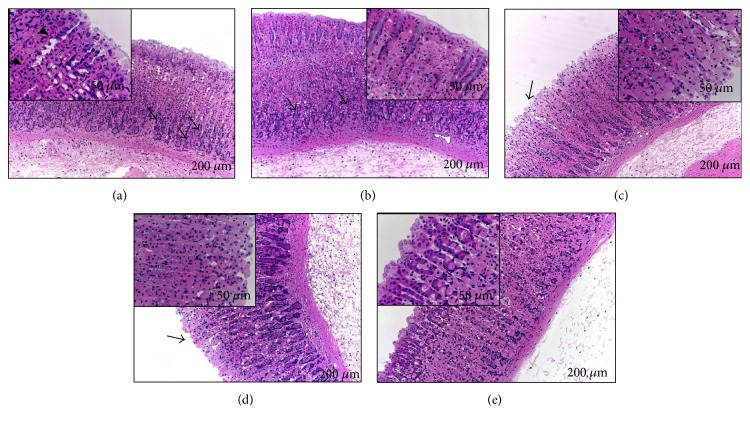
Histological analysis of the gastric mucosa of animals submitted to the induction of ulcer with ethanol. Animals were pretreated with 1% Tween 80 aqueous solution: injured control (a), lansoprazole (b), and SpHE 12.5 mg/kg (c), 25 mg/kg (d), and 50 mg/kg (e). Observed in (a): disorganization of the columnar simple epithelium in the gastric pits (enlarged detail, arrowhead) and gastric glands (arrows), congestion of the blood capillaries (enlarged detail, asterisk), and necrosis of the gastric mucosa cells (arrowhead); in (b): columnar simple epithelium in the gastric pits (enlarged detail) and well-preserved gastric glands (arrows); in (c): poorly preserved gastric mucosa, exfoliations of the columnar simple epithelium, and necrosis of the superficial epithelium (enlarged detail); and in (d) and (e): well-preserved gastric mucosa (enlarged detail).

**Figure 10:**
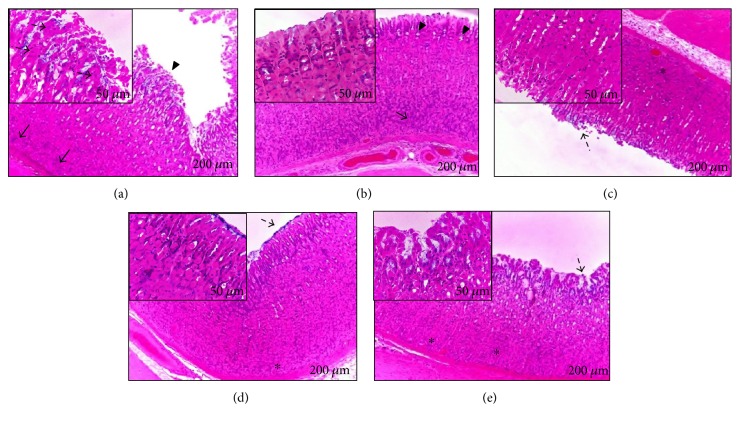
Histological analysis of the gastric mucosa of animals submitted to the induction of ulcer with NSAID (indomethacin). Animals were pretreated with 1% Tween 80 aqueous solution: injured control (a), ranitidine (b), and SpHE 12.5 mg/kg (c), 25 mg/kg (d), and 50 mg/kg (e). Observed in (a): disorganization of the gastric glands (arrows) and of the columnar simple epithelium in the gastric pits (enlarged detail, arrowhead) with cellular necrosis (dashed arrow); in (b): columnar simple epithelium of the pits (enlarged detail) and well-preserved gastric glands (arrows); in (c): gastric mucosa with slight exfoliations of the columnar simple epithelium and unpreserved gastric mucosa (asterisk); in (d): preserved gastric mucosa (asterisk) with exfoliations of discrete columnar epithelium (enlarged detail, arrow); and in (e): preserved gastric mucosa (asterisk) and simple columnar simple epithelium (enlarged detail).

Histological analysis of the gastric mucosa of animals with ulcers induced by HCl/ethanol (Figure 8), absolute ethanol (Figure 9), and NSAID (Figure 10) revealed disorganization of the columnar simple epithelium in the gastric pits and the gastric glands, as well as congestion of the blood capillaries. At a dose of 12.5 mg/kg SpHE, it is still possible to observe the poorly preserved gastric mucosa, presenting exfoliations of the simple columnar epithelium and necrosis of the superficial epithelium. However, at doses of 25 and 50 mg/kg SpHE and lansoprazole, a well-preserved gastric mucosa was observed.

### 3.8. Measurement of Gastric Mucosal Reduced Glutathione (GSH)

**Figure 11:**
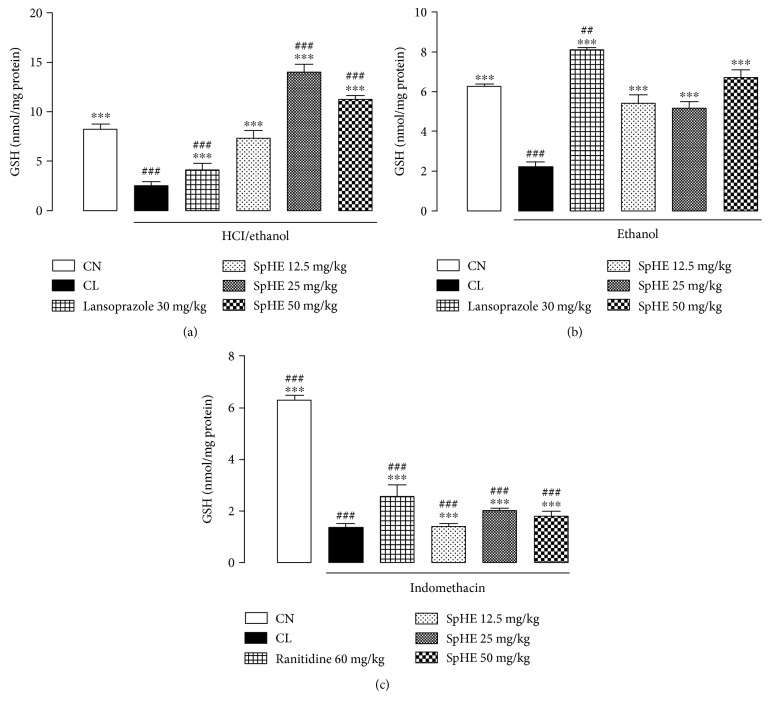
Effect of oral administration of SpHE on nonprotein sulfhydryl group (GSH) concentrations in the gastric mucosa of the animals submitted to models of induction of acute ulcers by HCl/ethanol (a), ethanol (b), and NSAID (c). The experimental groups received 1% Tween 80 aqueous solution (injured control (CL)). Results are expressed as mean ± SD (*n* = 5). ANOVA followed by Tukey's test, ^∗∗∗^*p* < 0.001 when compared with CL; ^##^*p* < 0.01; ^###^*p* < 0.001 when compared with noninjured control group (CN).

In the HCl/ethanol model, it was possible to verify that the groups pretreated with SpHE at doses of 12.5, 25, and 50 mg/kg or lansoprazole showed high concentrations of GSH (7.32 ± 0.74, 14.01 ± 0.73, 11.17 ± 0.42, and 4.11 ± 0.65 nmol/mg protein, resp.) when compared to the concentrations of the injured control, 2.45 ± 0.41 nmol/mg protein (noninjured control group = 8.22 ± 0.83 nmol/mg protein) (Figure 11(a)). In the absolute ethanol model, groups pretreated with SpHE (12.5, 25, and 50 mg/kg) or lansoprazole (30 mg/kg) also had high concentrations of GSH (5.42 ± 0.89, 5.12 ± 0.92, 6.67 ± 0.94, and 8.07 ± 0.34 nmol/mg protein, resp.), when compared to those of the injured control = 2.18 ± 0.59 nmol/mg protein (noninjured control group = 6.26 ± 0.20 nmol/mg protein) (Figure 11(b)). In Figure 11(c), it can be observed that similar results were obtained in the NSAID model, where the groups receiving ranitidine (60 mg/kg) or SpHE (12.5, 25, and 50 mg/kg) had higher GSH concentrations (2.52 ± 0.4, 1.41 ± 0.07, 2.03 ± 0.06, and 1.77 ± 0.19 nmol/mg protein, resp.) when compared to the injured control, 1.34 ± 0.16 nmol/mg protein (noninjured control group = 6.26 ± 0.20 nmol/mg protein). These results suggest a significant antioxidant activity.

### 3.9. Measurement of Gastric Mucosal Tumor Necrosis Factor (TNF-*α*)

**Figure 12:**
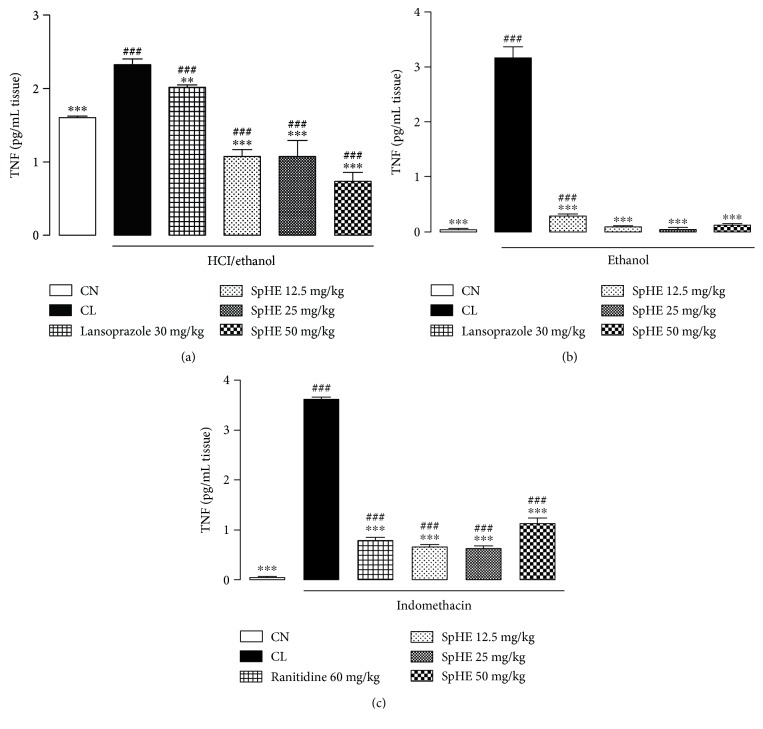
Effect of oral administration of SpHE on tumor necrosis factor alpha (TNF-*α*) concentrations in the gastric mucosa of the animals submitted to models of induction of acute ulcers by HCl/ethanol (a), ethanol (b), and NSAID (c). The experimental groups received 1% Tween 80 aqueous solution (injured control (CL)). Results are expressed as mean ± SD (*n* = 5). ANOVA followed by Tukey's test, ^∗∗^*p* < 0.01; ^∗∗∗^*p* < 0.001 when compared with CL; ^###^*p* < 0.001 when compared with noninjured control group (CN).

As shown in Figure 12(a), pretreatment of HCl/ethanol-injected mice with lansoprazole or SpHE (12.5, 25, and 50 mg/kg) caused a significant decrease in TNF-*α* (13, 54, 54, and 69%, resp.) compared to that of CL mice. Similar results were obtained for the ethanol model where groups pretreated with SpHE (12.5, 25, and 50 mg/kg) or lansoprazole also experienced a significant reduction in TNF-*α* concentrations (by 98, 99, 97, and 91%, resp.), when compared to those of the CL group (Figure 12(b)). As shown in Figure 12(c), similar results were obtained in the NSAID model, where the groups receiving ranitidine (60 mg/kg) or SpHE (12.5, 25, and 50 mg/kg) had a reduction in TNF-*α* concentrations (by 78, 82, 83, and 69%, resp.) when compared to those of the CL group.

### 3.10. Measurement of Gastric Mucosal NO

**Figure 13:**
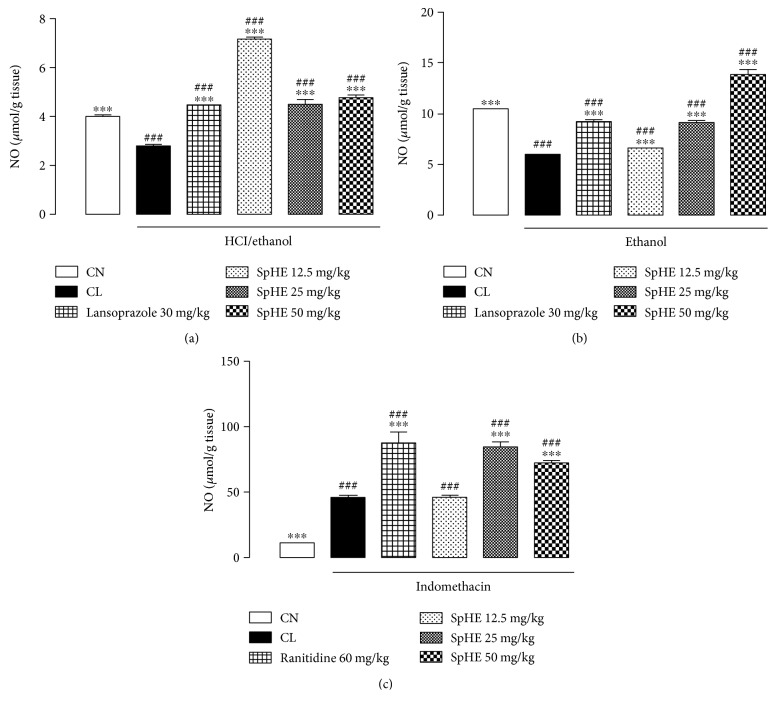
Effect of oral administration of SpHE on nitric oxide (NO) concentrations in the gastric mucosa of the animals submitted to models of induction of acute ulcers by HCl/ethanol (a), ethanol (b), and NSAID (c). The experimental groups received 1% Tween 80 aqueous solution (injured control (CL)). Results are expressed as mean ± SD (*n* = 5). ANOVA followed by Tukey's test, ^∗∗∗^*p* < 0.001 when compared with CL; ^###^*p* < 0.001 when compared with noninjured control group (CN).

In this model, it was possible to verify that the groups pretreated with SpHE at doses of 12.5, 25, and 50 mg/kg, or lansoprazole, showed high concentrations of NO (7.14 ± 0.06, 4.49 ± 0.18, 4.75 ± 0.08, and 4.47 ± 0.01 *μ*mol/g tissue, resp.) relative to the concentrations of the injured control, 2.78 ± 0.03 *μ*mol/g tissue (noninjured control group = 4.00 ± 0.01 *μ*mol/g tissue) (Figure 13(a)). In the ethanol model, groups pretreated with SpHE (12.5, 25, and 50 mg/kg) or lansoprazole (30 mg/kg) also had high concentrations of NO (6.59 ± 0.01, 9.14 ± 0.01, 13.75 ± 0.58, and 9.18 ± 0.01 *μ*mol/g tissue, resp.) when compared to the concentrations of the CL, 5.88 ± 0.01 *μ*mol/g tissue (noninjured control group = 10.39 ± 0.01 *μ*mol/g tissue) (Figure 13(b)). As shown in Figure 13(c), similar results were obtained in the NSAID model, where the groups receiving ranitidine (60 mg/kg) or SpHE (12.5, 25, and 50 mg/kg) had higher NO concentrations (87.04 ± 8.50, 45.63 ± 0.14, 84.14 ± 3.61, and 71.49 ± 0.82 *μ*mol/g tissue, resp.) than those of the CL group, 45.58 ± 1.22 *μ*mol/g tissue (noninjured control group = 10.39 ± 0.01 *μ*mol/g tissue). In the HCl/ethanol- and absolute ethanol-induced gastric mucosal injury models, gastric NO level was significantly lower in the CL group than in the CN group. In contrast, gastric mucosal NO level was significantly higher in the CL group than in the CN group in the NSAID-induced gastric injury model.

## 4. Discussion

Studies with *S. purpurea* are scarce and limited to fruits and gum exudates [27, 28, 30]. A study by Omena et al. [29] showed that the ethanol extract of the seeds and peel of *S. purpurea* had a high concentration of phenolic compounds and remarkable antioxidant activity. Another study showed the larvicidal activity of *S. purpurea* leaves in *Aedes aegypti* [47]. Methanolic extracts of *S. purpurea* bark were active against strains of *Staphylococcus aureus*, *Staphylococcus epidermidis*, *Pseudomonas aeruginosa*, *Bacillus cereus*, and *Escherichia coli* [22].

Thus, to the best of our knowledge, this study was the first to evaluate the phytochemical and pharmacological properties of the leaves of *S. purpurea* that are widely used in traditional medicine. A phytochemical screening was performed via TLC that revealed the marked presence of flavonoids in the extract. In addition to this result, the chromatograms of SpHE showed peaks caused by caffeic acid and epigallocatechin (Figure 1). Epigallocatechin has high antioxidant and antiulcerogenic capacities [48]. Similarly, caffeic acid (3,4-dihydroxycinnamic acid) is recognized for its remarkable antioxidant capacities *in vitro* by sequestering free radicals and *in vivo* by increasing the concentrations of endogenous antioxidants and preventing oxidation [49, 50].

Based on these findings, we sought to investigate the presence of phenolic compounds and the *in vitro* antioxidant activities of SpHE. A considerable quantity of phenolic compounds was found in SpHE. These compounds are widely distributed in nature and are considered to be potent antioxidants, both for their ability to donate hydrogen or electrons and for the stable intermediate radicals they create that prevent lipid oxidation [38].

The results of the four SpHE antioxidant activity assays (phosphomolybdenum, ABTS, FRAP, and DPPH) were also satisfactory. Studies indicate that there is no single method capable of quantitatively and accurately evaluating antioxidant properties. Rather, as these methodologies differ in their mechanisms of action, they are considered complementary in the study of the antioxidant potential of plants [51, 52]. The results of the *in vitro* antioxidant assays indicate that the hexanic extract of *S. purpurea* contains classes of bioactive compounds of interest with antiulcerogenic activities.

To investigate the antiulcerogenic activity promoted by SpHE, models of induced ulcers were obtained using acidified ethanol, absolute ethanol, and an NSAID (indomethacin). These models are widely used and well described in the literature [53, 54].

In the HCl/ethanol model, hydrochloric acid is responsible for causing severe damage to the gastric mucosa, enhancing the effect of ethanol. Ethanol produces necrotic lesions as a result of direct action on the mucosa and reduces defense factors, such as bicarbonate secretion and mucus production [55].

The model of ulcers in rats induced by indomethacin, a nonselective COX inhibitor, facilitates a more specific investigation into the cytoprotective potential and antisecretory capacity of an extract, since the physiopathology of this lesion involves gastric acid secretion and prostaglandin synthesis [56–58]. The indiscriminate use of NSAIDs causes damage to the gastric mucosa locally, specifically when these substances are chemically associated with the phospholipid layer, and cause the rupture of the mucosal surface. Furthermore, the damage is systemic when the COX enzymes are inhibited [56, 57, 59].

However, different doses of SpHE significantly reduced macroscopic lesions caused by acidified ethanol in mice (Figures 2 and 3), absolute ethanol in rats (Figures 4 and 5), and NSAID in rats (Figures 6 and 7), and the histopathological analyzes confirm these findings (Figures 8, 9, and 10).

The injury caused by the harmful agents was also associated with oxidative stress and the depletion of glutathione levels in the gastric mucosa. Glutathione is considered to be the most abundant low molecular weight cellular thiol and may present as GSH [60]. In this study, it was possible to verify that the groups pretreated with SpHE exhibited high concentrations of GSH in the three experimental models (Figure 11), which suggests a significant antioxidant activity.

Tumor necrosis factor is an important mediator of the acute inflammatory response and is involved in the apoptosis of gastric mucosal cells that are damaged by different agents. The administration of ethanol or indomethacin activates the innate immune system and promotes increased levels of TNF-*α* in the gastric tissue [54, 61, 62], as evidenced in the injured control. However, a significant decrease in TNF-*α* concentration in the groups pretreated with SpHE, as shown in Figure 12, was observed.

Nitric oxide is a liposoluble and unstable gas generated from the action of NO synthase (NOS) [63]. There are at least three isoforms of this enzyme in the human organism: neuronal NO synthase (nNOS), which is expressed in the central and peripheral nervous system; endothelial NO synthase (eNOS), localized in endothelial cells; and inducible NO synthase (iNOS), which produces NO at high concentrations and is present in macrophages, neutrophils, vascular cells, and endothelial cells [57, 64, 65].

In the ulcer, NO can present a dual effect, both antiulcerogenic and ulcerogenic, depending on the isoform involved. The constitutive isoforms eNOS and nNOS produce low amounts of NO and are responsible for repair and healing of ulcers, increased mucosal blood flow, and angiogenesis [57, 66]. Thus, in this work, on the HCl/ethanol- and absolute ethanol-induced gastric mucosal injury models, gastric NO level was significantly lower in the CL group than in the CN group (Figure 13), suggesting that this low concentration of NO is due to the expression of the constitutive isoforms and therefore is protective of the gastric mucosa. In contrast, in the NSAID-induced gastric injury model (Figure 13), gastric mucosal NO level was significantly higher in the CL group than in the CN group. According to literature data, when generated from the iNOS isoform, NO can promote the formation of the lesion by stimulating apoptosis, which depends on the redox state of the tissue and the amount and duration of the enzymatic expression [57, 66]. Thus, in this model, it is suggested that SpHE had an antiulcerogenic effect by mechanisms that are independent of NO, but it involves the participation of GSH and reduction of TNF-*α*.

In conclusion, according to the results of the present study, it is possible to conclude that SpHE possesses high antioxidant capacities, possibly due to the presence of the major compounds identified, such as caffeic acid and epigallocatechin; expressive antiulcer activities in the models of acute gastric lesions, with the involvement of GSH and the ability to reduce TNF-*α* in the models, besides the participation of NO in the HCl/ethanol and ethanol models.

## Figures and Tables

**Table 1:** Percentage of sequestration of DPPH radicals in different concentrations of *S. purpurea*.

SpHE (*μ*g/mL)	DPPH (RSA%)
1000	39.30 ± 0.67
500	17.39 ± 0.46
250	9.83 ± 0.13
125	7.92 ± 0.65
62.5	1.68 ± 0.17
31.25	1.04 ± 0.32

Results are expressed as mean ± SD (*n* = 3).

## References

[1] Caldas G. F. R., Oliveira A. R. S., Araújo A. V. (2014). Gastroprotective and ulcer healing effects of essential oil of *Hyptis martiusii* Benth. (Lamiaceae). *PLoS One*.

[2] Bansal V. K., Goyal S. K., Goswami D. S., Singla S., Rahar S., Kumar S. (2009). Herbal approach to peptic ulcer disease – REVIEW. *Journal Bioscience Technolgy*.

[3] Bertleff M. J., Lange J. F. (2010). Perforated peptic ulcer disease: a review of history and treatment. *Digestive Surgery*.

[4] Lau J. Y., Sung J., Hill C., Henderson C., Howden C. W., Metz D. C. (2011). Systematic review of the epidemiology of complicated peptic ulcer disease: incidence, recurrence, risk factors and mortality. *Digestion*.

[5] Thorsen K., Soreide J. A., Kvaloy J. T., Glomsaker T., Søreide K. (2013). Epidemiology of perforated peptic ulcer: age- and gender-adjusted analysis of incidence and mortality. *World Journal Gastroenterology*.

[6] Zelickson M. S., Bronder C. M., Johnson B. L. (2011). *Helicobacter pylori* is not the predominant etiology for peptic ulcers requiring operation. *The American Surgeon*.

[7] Bansal V. K., Goel R. K. (2012). Gastroprotective effect of *Acacia nilotica* young seed lesspod extract: role of polyphenolic constituents. *Asian Pacific Journal of Tropical Medicine*.

[8] Alqasoumi S., Al-Sohaibani M., Al-Howiriny T., Al-Yahya M., Rafatullah S. (2009). Rocket *Eruca sativa*: a salad herb with potential gastric anti-ulcer activity. *World Journal Gastroenterology*.

[9] Prabha T., Dorababu M., Goel S. (2009). Effect of methanolic extract of *Pongamia pinnata* Linn seed on gastro-duodenal ulceration and mucosal offensive and defensive factors in rats. *Indian Journal of Experimental Biology*.

[10] Oyagi A., Ogawa K., Kakino M., Hara H. (2010). Protective effects of a gastrointestinal agent containing Korean red ginseng on gastric ulcer models in mice. *BMC Complementary and Alternative Medicine*.

[11] Alrashdi A. S., Salama S. M., Alkiyumi S. S. (2012). Mechanisms of gastroprotective effects of ethanolic leaf extract of *Jasminum sambac* against HCl/ethanol-induced gastric mucosal injury in rats. *Evidence-Based Complementary and Alternative Medicine*.

[12] Brzozowski T., Ptak-Belowska A., Kwiecien S. (2012). Novel concept in the mechanism of injury and protection of gastric mucosa: role of rennin-angiotensin system and active metabolites of angiotensin. *Current Medicinal Chemistry*.

[13] Muthuraman A., Sood S. (2010). Antisecretory, antioxidative and antiapoptotic effects of montelukast on pyloric ligation and water immersion stress induced peptic ulcer in rat. *Prostaglandins, Leukotrienes and Essential Fatty Acids*.

[14] DeVault K. R., Talley N. J. (2009). Insights into the future of gastric acid suppression. *Natural Reviews Gastroenterology and Hepatology*.

[15] Bardi D., Khan M. S., Sabri S. (2011). Anti-ulcerogenic activity of *Typhonium flagelliforme* aqueous leaf extract against ethanol induced gastric mucosal injury in rats. *Scientific Research and Essays*.

[16] Rabelo A. S., Oliveira I. D., Guimarães A. G. (2013). Antinociceptive, anti-inflammatory and antioxidant activities of aqueous extract from *Remirea maritima* (Cyperaceae). *Journal of Ethnopharmacology*.

[17] Engels C., Gräter D., Esquivel P., Jiménez V. M., Gänzle M. G., Schieber A. (2012). Characterization of phenolic compounds in jocote (*Spondias purpurea* L.) peels by ultra high-performance liquid chromatography/electrospray ionization mass spectrometry. *Food Research International*.

[18] Wannan B. S. (2006). Analysis of generic relationships in Anacardiaceae. *Blumea- Biodiversity, Evolution and Biogeography of Plants*.

[19] Bachelier J. B., Endress P. K. (2009). Comparative floral morphology and anatomy of Anacardiaceae and Burseraceae (Sapindales), with a special focus on gynoecium structure and evolution. *Botanical Journal of the Linnean Society*.

[20] Miller A., Schaal B. (2005). Domestication of a Mesoamerican cultivated fruit tree, *Spondias purpurea*. *Proceedings of the National Academy of Sciences*.

[21] Caceres A., Cano O., Samayoa B., Aguilar L. (1990). Plants used in Guatemala for the treatment of gastrointestinal disorders. 1. Screening of 84 plants against enterobacteria. *Journal of Ethnopharmacology*.

[22] Agra M. F., Freitas P. F., Barbosa-Filho J. M. (2007). Synopsis of the plants known as medicinal and poisonous in northeast of Brazil. *Brazilian Journal of Pharmacognosy*.

[23] Cabral B., Siqueira E., Bitencourt M. A. (2006). Phytochemical study and anti-inflammatory and antioxidant potential of *Spondias mombin* leaves. *Brazilian Journal of Pharmacognosy*.

[24] Almeida M. M. B., Sousa P. H. M., Arriaga A. M. C. (2011). Bioactive compounds and antioxidant activity of fresh exotic fruits from northeastern Brazil. *Food Research International*.

[25] Sakunpak A., Panichayupakaranant P. (2012). Antibacterial activity of Thai edible plants against gastrointestinal pathogenic bacteria and isolation of a new broad spectrum antibacterial polyisoprenylated benzophenone, chamuangone. *Food Chemistry*.

[26] Lemos T. L. G., Nogueira P. C. L., Alencar J. W., Craveiro A. A. (1995). Composition of the leaf oils of four *Spondias* species from Brazil. *Journal of Essential Oil Research*.

[27] Murillo E., Meléndez-Martínez A. J., Portugal F. (2010). Screening of vegetables and fruits from Panama for rich sources of lutein and zeaxanthin. *Food Chemistry*.

[28] Ceva-Antunes P. M., Bizzo H. R., Silva A. S., Carvalho C. P. S., Antunes O. A. C. (2006). Analysis of volatile composition of siriguela (*Spondias purpurea* L.) by solid phase microextraction (SPME). *LWT - Food Science and Technology*.

[29] Omena C. M. B., Valentim I. B., Guedes G. S. (2012). Antioxidant, anti-acetylcholinesterase and cytotoxic activities of ethanol extracts of peel, pulp and seeds of exotic Brazilian fruits: antioxidant, anti-acetylcholinesterase and cytotoxic activities in fruits. *Food Research International*.

[30] Martínez M., Pinto G. L., González M. B., Herrera J., Oulyadi H., Guilhaudis L. (2008). New structural features of *Spondias purpurea* gum exudates. *Food Hydrocolloids*.

[31] Wagner H., Bladt S. (1996). *Plant Drug Analysis - A Thin Layer Chromatography Atlas*.

[32] Brasseur T., Angenot L. (1986). Un reactif de choix pour la revelation des flavonoîdes: Le melange diphenylborate d’aminoethanol -PEG 400. *Bulletin Liaison Groupe Polyphenols*.

[33] Roberts E. A. H., Cartwright R. A., Oldschool M. (1957). Phenolic substances of manufactured tea. I. Fractionation and paper chromatography of water-soluble substances. *Journal of the Science of Food and Agriculture*.

[34] Li H. B., Wong C. C., Cheng K. W., Chen F. (2008). Antioxidant properties in vitro and total phenolic contents in methanol extracts from medicinal plants. *LWT - Food Science and Technology*.

[35] Prieto P., Pineda M., Aguilar M. (1999). Spectrophotometric quantitation of antioxidant capacity through the formation of a phosphomolybdenum complex: specific application to the determination of vitamin E. *Analitical Biochemistry*.

[36] Benzie I. F., Strain J. J. (1996). The ferric reducing ability of plasma (FRAP) as a measure of “antioxidant power”: the FRAP assay. *Analitical Biochemistry*.

[37] Re R., Pellegrini N., Proteggente A., Pannala A., Yang M., Rice-Evans C. (1999). Antioxidant activity applying an improved ABTS radical cation decolorization assay. *Free Radical Biology & Medicine*.

[38] Brand-Williams W., Cuvelier M. E., Berset C. L. W. T. (1995). Use of a free radical method to evaluate antioxidant activity. *LWT - Food Science and Technology*.

[39] Mizui T., Douteuchi M. (1983). Effect of polyamines on acidified ethanol gastric-lesions in rats. *The Japanese Journal of Pharmacology*.

[40] Szelenyi I., Thiemer K. (1978). Distention ulcer as a model for testing of drugs for ulcerogenic side effects. *Archives of Toxicology*.

[41] Morimoto Y., Shimohara K., Oshima S., Sukamoto T. (1991). Effects of the new antiulcer agent KB-5492 on experimental gastric mucosal lesions and gastric mucosal defensive factors, as compared to those of teprenone and cimetidine. *The Japanese Journal of Pharmacology*.

[42] Djahanguri B. (1969). The production of acute gastric ulceration by indomethacin in the rat. *Scandinavian Journal of Gastroenterology*.

[43] Lemos A. J. J. M., Peixoto C. A., Teixeira A. C. C. (2014). Effect of the combination of metformin hydrochloride and melatonin on oxidative stress before and during pregnancy, and biochemical and histopathological analysis of the livers of rats after treatment for polycystic ovary syndrome. *Toxicology and Applied Pharmacology*.

[44] Almeida L. L., Teixeira A. C. C., Wanderley-Teixeira V. (2014). Histopathological analysis of the small intestine of pregnant rats exposed to sub-lethal doses of herbicides and treated with melatonin. *Experimental Pathology and Health Science*.

[45] Sedlak J., Lindsay R. H. (1968). Estimation of total protein bound and non protein sulfhydril groups in tissues with Ellman’s reagent. *Analitical Biochemistry*.

[46] Giustarini D., Rossi R., Milzani A., Dalle-Donne I. (2008). Nitrite and nitrate measurement by Griess reagent in human plasma: evaluation of interferences and standardization. *Methods in Enzymology*.

[47] Lima M. A., Oliveira F. F. M., Gomes G. A. (2011). Evaluation of larvicidal activity of the essential oils of plants species from Brazil against *Aedes aegypti* (Diptera: Culicidae). *African Journal of Biotechnology*.

[48] Hamaishi K., Kojima R., Ito M. (2006). Anti-ulcer effect of tea catechin in rats. *Biological and Pharmaceutical Bulletin*.

[49] Janbaz K. H., Saeed S. A., Gilani A. H. (2004). Studies on the protective effects of caffeic acid and quercetin on chemical-induced hepatotoxicity in rodents. *Phytomedicine*.

[50] Sun-Waterhouse D., Zhou J., Miskelly G. M., Wibisono R., Wadhwa S. S. (2011). Stability of encapsulated olive oil in the presence of caffeic acid. *Food Chemistry*.

[51] Rebaya A., Belghith S. I., Baghdikian B. (2015). Total phenolic, total flavonoid, tannin content, and antioxidant capacity of *Halimium halimifolium* (Cistaceae). *Journal of Applied Pharmaceutical Science*.

[52] Prior R. L., Wu X., Schaich K. (2005). Standardized methods for the determination of antioxidant capacity and phenolics in foods and dietary supplements. *Journal of Agricultural and Food Chemistry*.

[53] Suhaimy N. W. I., Azmi A. K. N., Mohtarrudin N. (2017). Semipurified ethyl acetate partition of methanolic extract of *Melastoma malabathricum* leaves exerts gastroprotective activity partly via its antioxidant-antisecretory-anti-inflammatory action and synergistic action of several flavonoid-based compounds. *Oxidative Medicine and Cellular Longevity*.

[54] Almasaudi S. B., El-Shitany N. A., Abbas A. T. (2016). Antioxidant, anti-inflammatory, and antiulcer potential of manuka honey against gastric ulcer in rats. *Oxidative Medicine and Cellular Longevity*.

[55] Ateufack G., Mokam E. C. D., Mbiantcha M., Feudjio R. B., David N., Kamanyi A. (2015). Gastroprotective and ulcer healing effects of *Piptadeniastrum africanum* on experimentally induced gastric ulcers in rats. *BMC Complementary and Alternative Medicine*.

[56] Wallace J. L. (2008). Prostaglandins, NSAIDs, and gastric mucosal protection: why doesn’t the stomach digest itself?. *Physiological Reviews*.

[57] Musumba C., Pritchard D. M., Pirmohamed M. (2009). Cellular and molecular mechanisms of NSAID-induced peptic ulcers. *Alimentary Pharmacology and Therapeutics*.

[58] Adinortey M. B., Ansah C., Galyuon I., Nyarko A. (2013). In vivo models used for evaluation of potential antigastroduodenal ulcer agents. *Ulcers*.

[59] Lichtenberger L. M., Zhou Y., Dial E. J., Raphael R. M. (2006). NSAID injury to the gastrointestinal tract: evidence that NSAIDs interact with phospholipids to weaken the hydrophobic surface barrier and induce the formation of unstable pores in membranes. *Journal of Pharmacy and Pharmacology*.

[60] Jozefczak M., Remans T., Vangronsveld J., Cuypers A. (2012). Glutathione is a key player in metal-induced oxidative stress defenses. *International Journal of Molecular Science*.

[61] Nakashita M., Suzuki H., Miura S. (2013). Attenuation of acetic acid-induced gastric ulcer formation in rats by glucosylceramide synthase inhibitors. *Digestive Diseases and Sciences*.

[62] Salga M. S., Ali H. M., Abdulla M. A. (2012). Gastroprotective activity and mechanism of novel dichlorido-zinc(II)-4-(2-(5-methoxybenzylideneamino)ethyl)piperazin-1-iumphenolate complex on ethanol-induced gastric ulceration. *Chemico-Biological Interactions*.

[63] Luo J., Chen A. F. (2005). Nitric oxide: a newly discovered function on wound healing. *Acta Pharmacologica Sinica*.

[64] Pacher P., Beckman J. S., Liaudet L. (2007). Nitric oxide and peroxynitrite in health and disease. *Physiological Reviews*.

[65] Nageswararao K. B., Lakshmi K., Ramakrishna R. (2011). Nitric oxide: a novel therapeutic target. *International Journal of Pharmaceutical Science Research*.

[66] Calatayud S., Barrachina D., Esplugues J. V. (2001). Nitric oxide: relation to integrity, injury, and healing of the gastric mucosa. *Microscopy Resarch and Techique*.

